# Characterization of three-nucleate *Rhizoctonia* AG-E based on their morphology and phylogeny

**DOI:** 10.1038/s41598-023-44448-1

**Published:** 2023-10-13

**Authors:** Ewa Moliszewska, Dagna Maculewicz, Hanna Stępniewska

**Affiliations:** 1 Faculty of Natural Sciences and Technology, Institute of Environmental Engineering and Biotechnology, University of Opole, Opole, Poland; 2 Faculty of Health Sciences, Institute of Health Sciences, University of Opole, Opole, Poland; 3 Department of Forest Ecosystems Protection, Faculty of Forestry, University of Agriculture in Krakow, Kraków, Poland

**Keywords:** Environmental sciences, Microbiology techniques, Microscopy, Biodiversity, Microbiology, Applied microbiology, Environmental microbiology, Fungi

## Abstract

The genus *Rhizoctonia* has been classified into two main groups according to the number of nuclei. Binucleate *Rhizoctonia* strains have two nuclei in each cell, whereas multinucleate *Rhizoctonia* fungi were observed to have a variable number of nuclei ranging from 4 to 16 in each cell. In the study, twelve Polish isolates were tested. According to ITS1-5,8S-ITS2 rDNA sequences, the isolates were classified in the AG-E. Their affiliation to AG was confirmed by anastomosis reactions with tester isolates. The number of nuclei was counted with DAPI staining under a fluorescent microscope, and the diameter of the hyphae was also measured. Not all AG-E isolates had the same number of nuclei in their cells: one group among these fungi produced cells with a diverse number of nuclei, usually 3; however, this number ranged from 2 to 4, making the average number of nuclei close to 3. It can be assumed that all isolates with three nuclei belong to this group, which may greatly facilitate the preliminary identification of trinucleate isolates of *Rhizoctonia* spp. belonging to AG-E. Based on these characters, we call these isolates AG-E-3n isolates. The thiamine requirement is not helpful in classifying and describing the AG-E strains.

## Introduction

The genus *Rhizoctonia* was first described in 1815. It is a very diverse group of saprotrophic, pathogenic, and mycorrhizal soil fungi. Pathogenic isolates usually cause damage to underground parts of the plant, but sometimes the disease symptoms are also visible on the aerial parts, or destruction of the whole plant occurs. They can also function perfectly as saprotrophs^1^. Within this genus, many mycorrhizal species have also been identified, mainly with plants from the Orchidaceae family^2,3^. The diversity of trophic preferences of isolates of *Rhizoctonia* spp. causes quality and yield losses in many plant species—cereals, ornamentals, vegetables, trees, and others. *Rhizoctonia* spp. has been reported in more than 40 countries, but it is possible that they occur worldwide. Population composition is closely related to climate and species composition in a particular area^4^.

Within the genus *Rhizoctonia*, the primary classification is based on the number of nuclei in cells and the diameter of hyphae, usually between 3 and 7 µm^5^. Fungi that have 2 nuclei, the average for this genus, are considered binucleate *Rhizoctonia* (BNR = binucleate *Rhizoctonia*) with the *Ceratobasidium* spp. sexual morph. BNR isolates show pathogenic properties against a wide range of plant species. They can cause various types of damage, such as damping-off and root rot. They may also serve as organisms beneficial to plants, acting as mycorrhizal or protective fungi^3,6^. Members of the *Rhizoctonia* genus are basically tested according to thiamine requirement, which leads to indicate some groups as thiamine-auxotrophic or prototrophic. Thiamine plays a stimulative role in auxotrophic organisms, leading to even tenfold growth or greater. Such features were indicated for the members of some anastomosis groups (AG) e.g. *R. solani* AG-1 or AG-2-2IIIB, AG-2-2IV, and AG-5^7,8^.

Within the same anastomosis groups (AG), there are usually isolates with similar trophic preferences, but the basis for determining group membership is to examine the nature of the reaction between the hyphae of the examined and tester isolates. Molecular analysis of the ITS1-5,8S-ITS2 regions of ribosomal DNA and comparison with the sequence database also allows categorization to the particular AGs and is currently considered the best *Rhizoctonia* classification method^9,10^. Classical hyphal fusion is a time-consuming and labor-intensive method. Sometimes its effects are unreliable because some isolates of the same group do not always anastomose or anastomose at a low frequency. It also happens that isolates from different groups anastomose with each other^11^.

The BNR has been divided into 19 anastomosis groups, from AG-A to AG-W, based on the anastomosis reactions between their hyphae^9,10,12^. The division into independent anastomosis groups within the BNR group was proposed by Ogoshi et al.^13,14^ and Burpee et al.^15^. Ogoshi grouped his isolates into AG-A through AG-Q, while the North American isolates of Burpee were grouped in CAG-1 through CAG-7. Five groups of CAGs turned out to have their counterparts in the AGs proposed by Ogoshi. However, the Ogoshi classification is most often used. The members of the two CAGs were not described by Ogoshi, and they were finally assigned to AG-R (CAG-5) and AG-S (CAG-7). After a more detailed examination, CAG-3 and CAG-6 turned out to be the same group (AG-E), although characteristic anastomosing reactions between them had not been previously observed^13^. The anastomosis division given by Ogoshi is successfully used up to this day, and it is constantly expanded and modified. Hyakumachi and co-authors^16^ identified two new, AG-T and AG-U, anastomosis groups within the BNR. Representatives of these groups were isolated from miniature roses grown in Japan. During that time some anastomosis groups were excluded, e.g. AG-J was excluded because it turned out that the fungi belonging to that group formed clamp connections, while AG-M is currently not isolated and does not occur in any of the known collections^11,17^. AG -T already no longer exists because its isolates anastomosed with AG-A isolates, so the group was included in AG-A^11^. Analysis of the ITS rDNA sequence of the AG-N group (there is only one sequence available in the NCBI database) showed 61–72% similarity to the other BNR groups, while the sequences of the ITS regions of the other groups are typically similar at the level of 75–95%^11^. Phylogenetic analysis shows that AG-F, AG-E, AG–P, AG-R, and AG–S arise separately from other BNR clades in dendrograms based on ITS1-5,8S-ITS2 rDNA sequences and are genetically more closely related to *R. solani* than to BNRs^9,18^.

However, despite BNRs occurring worldwide, there are not many articles discussing their taxonomically useful features. Hietala et al.^19^ and Otero et al.^2^ published uninucleate isolates of *Ceratobasidium* sp. Moliszewska^20^ mentioned trinucleate BNRs isolated from sugar beet seedlings’ roots. They were obtained from roots, together with multinucleate *R. solani* and other BNRs (AG-K). She pointed out that some of the isolates showed a distinct trinucleate state. A piece of similar information was mentioned by Adams and Butler^21^. They noticed the presence of trinucleate isolates of *Rhizoctonia* spp. among other isolates. These isolates did not match other *Rhizoctonia* strains they tested. However, they did not continue their investigation.

According to our observations, the division of *Rhizoctonia* spp. based on the number of nuclei in binucleate and multinucleate strains may not be obvious. Thus, the aim of this study was to examine a group of previously classified BNR isolates with an unclear two- or three-nuclei stage.

## Materials and methods

### Sample collection and fungal isolation

The study was based on a collection of fungi of the genus *Rhizoctonia* isolated from woody and herbaceous plants, as well as sugar beet seedlings from southern Poland (Table 1). The isolates came from the collection of the Department of Forest Ecosystems Protection, the University of Agriculture in Krakow (12 of HS isolates; collection of Hanna Stępniewska Ph.D.) (Table 1). The tester binucleate *Rhizoctonia* spp. isolates came from the collection of prof. Mitsuro Hyakumachi of Gifu University (Japan), courtesy of Takeshi Toda, Ph.D. of Akita Prefectural University in Akitashi (Japan) (Table 2). Isolation was performed according to classical phytopathological and mycological procedures. The collected research material was stored on PDA slants at 4 °C for immediate use. For long-term storage, cultures were prepared on wheat or millet grains and stored frozen at − 30 °C^22^.

**Table 1:** AG-E isolates collected from Poland and used in this study.

Isolate	Host plant	Location	Date of collection	AG	Collector	GeneBank Accession number
HS10	*Pinus sylvestris*	Dębica	1997	AG-E	H. Stępniewska	KX831947
HS21	*Betula pendula*	Złoty Potok	2001	AG-E	H. Stępniewska	KX831953
HS23	*P. sylvestris*	Niepołomice	2001	AG-E	H. Stępniewska	KX831955
HS26	*Lupinus luteus*	Dąbrowa Tarnowska	1995	AG-E	H. Stępniewska	KX831957
HS27	*L. luteus*	Dębica	1995	AG-E	H. Stępniewska	KX831958
HS29	*Vicia* sp.	Dębica	1995	AG-E	H. Stępniewska	KX831959
HS30	*Lupinus angustifolius*	Dębica	1995	AG-E	H. Stępniewska	KX831960
HS33	*P. sylvestris*	Dąbrowa Tarnowska	1997	AG-E	H. Stępniewska	KX831961
HS38	*P. sylvestris*	Dębica	1997	AG-E	H. Stępniewska	KX831963
HS45	*P. sylvestris*	Niepołomice	2010	AG-E	H. Stępniewska	KX831965
HS46	*P. sylvestris*	Niepołomice	2010	AG-E	H. Stępniewska	KX831966
HS47	*P. sylvestris*	Niepołomice	2010	AG-E	H. Stępniewska	KX831967

**Table 2:** Tester isolates of binucleate *Rhizoctonia.*

Anastomosis group	Isolate code	Origin	Host plant/source
AG-A (= AG-T)	AH-1	Japan	*Arachis hypogaea*
	C538	Japan	*Solanum tuberosum*
AG-Ba	C484	Japan	*Oryza sativa*
AG-Bb	C350	Japan	*O. sativa*
AG-C	Gs-1	Japan	soil
	C460	Japan	soil
AG-DI (AG-Q)	YC-SDS-1	Japan	*Zoysia japonica*
	BlG-WP-1	Japan	*Agrostis palustris*
AG-DII	OK-EF-1	Japan	*Z. japonica*
AG-DIII	KOU04-12FW	Japan	*Z. japonica*
AG-E	RH155	Japan	*Dactylis glomerata*
	Lu-1	Japan	*Linum usitatissimum*
AG-F	AH-6	Japan	*A. hypogaea*
AG-G	AH-9	Japan	*A. hypogaea*
AG-H	STC-11	Japan	soil
AG-I	AV-2	Japan	*Artemisia* sp.
	FKO-1-13	Japan	soil
AG-K	AC-1	Japan	*Allium cepa*
	55D45	Japan	*Beta vulgaris*
AG-L	FKO-2-26	Japan	soil
	FKO-2-20	Japan	soil
AG-O	FKO-6-2	Japan	soil
	FKO-2-11	Japan	soil
AG-P	C578	Japan	*Thea sinensis*
	C584	Japan	soil
AG-R	BN-37	USA	*Cucumis sativus*
AG-S	S5	USA	soil
AG-U	MWR-24	Japan	*Rosa hybrida*
	Rh29B	USA	*Rhododendron eriocarpum*

### Morphological characteristics, nuclear condition, and hyphae diameter

Isolates listed in Tables 1 and 2 were grown on Potato Dextrose Agar (PDA, Biomaxima) for 2 weeks and then the morphological appearance of mycelium (color and growth intensity), presence of zonation (present/absent), and sclerotia (+—poor, +  +—little, +  +  +—medium, +  +  +  +—abundant) were determined. Cultures that were grown on a PDA medium also were used to evaluate their growth in various temperatures (8, 22, 30 ± 1 °C). Results were shown as daily growth given in centimeters (cm/day). Young *Rhizoctonia* cultures (3–7 days old) grown on PDA were used to measure the hyphae diameter and number of nuclei per cell. Observations were made microscopically under the 40 × objective, and the diameter of the hyphae was measured on young and well-developed running hyphae, with 50 measurements for each isolate. The number of nuclei in young cells was counted after safranin O staining^23^ under a light microscope and DAPI (4′,6-diamidino-2-phenylindole) staining under a fluorescent microscope. The mean number of nuclei was determined based on 40 measurements.

### DNA Isolation and amplification

Isolates were grown in a liquid peptone medium (per liter: soy peptone—10 g, yeast extract—5 g, NaCl—10 g) for 1 week, after which the mycelium was centrifuged, dried, and stored at − 20 °C for further use. Isolation of genomic DNA was performed using the CTAB method^24,25^. DNA samples were stored at − 20 °C. Fragments of ITS1-5.8S-ITS2 rDNA were amplified with conventional primers ITS1 and ITS4^26^. PCR reactions consisted of 30 cycles, each with 1 min of denaturation at 93 °C, 1 min of hybridization at 57 °C, and 2 min of elongation at 72 °C. Initial denaturation was conducted for 2.5 min. at 93 °C and the final synthesis lasted for 10 min. at 72 °C (according to^27^). The products of PCR reactions were purified using an Exo-Sap kit and sequenced at Genomed (Poland). Sequences were compared with those deposited in GenBank using the BLAST tool^28^.

### Determination of anastomosis groups

**Figure 1:**
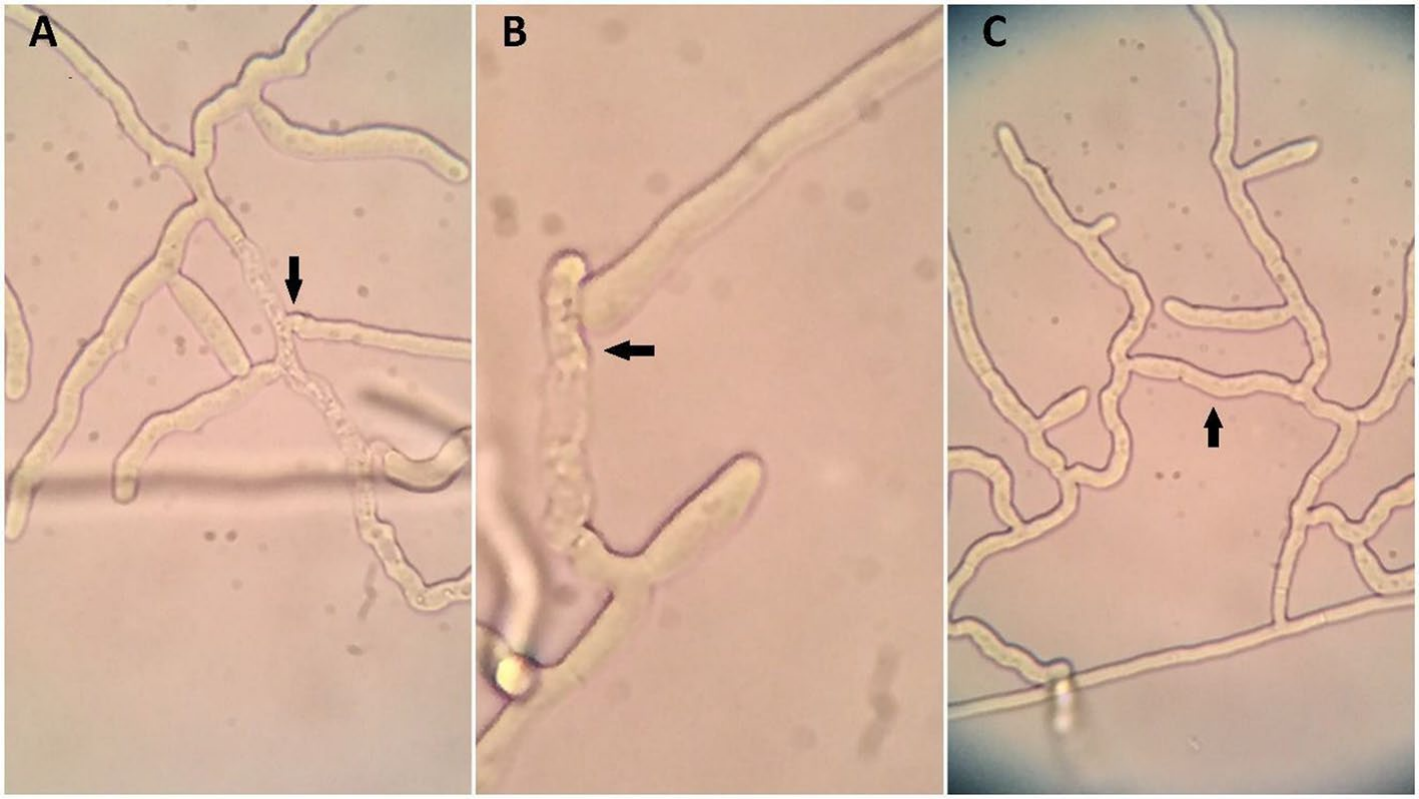
A, B—killing reaction (C2 fusion) between tested isolate and tester and C—self-fusion between hyphae of the same isolate (C3–perfect fusion) (phot. D. Maculewicz).

The initial determination of the AGs was done by comparison with the GenBank sequences. Subsequently, the results were verified according to the classical method of Kronland and Stanghellini^29^ and Moliszewska and Schneider^30^ with the testers described in Table 2. The type of reaction was determined according to the criteria given by Carling et al.^31^; at least 5 contact points showing a C2-type reaction (Fig. 1) were used to confirm AG; observations for each isolate were repeated at least twice. These tests were done for randomly selected isolates—HS27, HS29, HS30, HS38, HS46, HS47.

### Phylogenetic relationships

The phylogenetic relationships of our BNR were visualized using the dendrogram generated based on the ITS1-5.8S-ITS2 sequences. We used our BNRs’ sequences as well as sequences of various BNR anastomosis groups from the GenBank database (Table 3). Other sequences of *Rhizoctonia* AG-E isolates tested previously by Moliszewska^20^ were included in this research. The ITS1-5.8S-ITS2 rDNA sequence of *Athelia (Sclerotium) rolfsii* (isolate FSR052, accession number in GenBank AY684917) was used as an external group (according to^11^).

**Table 3:** Reference GenBank sequences used in phylogenetic analysis.

Isolate code	Accession no.	AG	Origin	Host plant/source
FSR-052	AY684917	*Athelia rolfsii* (outsource group)	nd*	nd
Rh228	KC782943	AG-A	Italy	soil
HLJ-RAB2	JX073670	AG-A	China	*Beta vulgaris* L. subsp*. vulgaris*
T-1	FR734288	AG-A	Turkey	*Nicotiana tabacum*, soil
Lu-5, MAFF 305,296	AB290018	AG-E	Japan	*Linum usitatissimum*
BN74	AF354083	CAG6, AG-E	USA	*Erigeron* sp.
nd	DQ279013	AG-E	nd	nd
RhCaGo-85	MW999187	AG-E	Sweden: Gothland	*Daucus carota*
C249	MZ395973	AG-E	USA	*Triticum aestivum*
OC-1 (= MAFF 305,299, = ATCC 38,676)	AB290019	AG-E	nd	nd
DB198	OM045908	AG-E	USA	*Phaseolus vulgaris*
HB1	KP662688	AG-K	China	*Beta vulgaris* L. subsp*. vulgaris*
AC-1	AB122145	AG-K	Japan	*Allium cepa*
WUF-ST-Rhb5	JQ859863	AG-K	Australia	*Fragaria* x *ananassa*
WUF-ST-RhT4-6	JQ859886	AG-B	Australia	*Fragaria* x *ananassa*
55D25	AB290021	AG-C	Japan	*Beta vulgaris* L. subsp.* vulgaris*
TAK-14KT	AB214367	AG-D	Japan	*Zoysia* sp.
SR-40	KF857549	AG-F	China	*Spinacia oleracea*
R25	AY927329	AG-G	Italy	*Fragaria* x *ananassa*
STC-9	AF354089	AG-H	Japan	soil
FKO-6–7	AB290022	AG-I	nd	nd
FKO-2-26, MAFF305324	AB196653	AG-L	Japan	soil
nd	DQ279045	AG-O	nd	nd
C-584	AB286938	AG-P	nd	nd
nd	DQ279061	AG-Q	nd	nd
J-04-7	DQ885781	AG-R	China	*Zingiber* sp.
RhMY071WCz2	HQ269819	AG-S	nd	*Azalea*, cv. ‘Gumpo’
BS-J-06-6-3	KM505159	AG-V	China	*Zingiber officinale*

*nd no determined.

The BLAST program^28^ was used to check whether homology existed between the isolates (value limit E > 10^–3^). Sequence comparison was performed with ClustalW^32^ and with MEGA X^33^ and then corrected according to the suggestions of Sharon et al.^11^. The phylogenetic tree was generated using ITS1-5.8S-ITS2 sequences rDNA with MEGA X^33^ by the Maximum likelihood method. The evolutionary history was inferred by using the Maximum Likelihood method and the Tamura-Nei model^34^. The tree with the highest log likelihood (− 5080,16) is shown. The percentage of trees in which the associated taxa clustered together is shown next to the branches. Initial tree(s) for the heuristic search were obtained automatically by applying Neighbor-Join and BioNJ algorithms to a matrix of pairwise distances estimated using the Tamura-Nei model, and then selecting the topology with superior log likelihood value. The proportion of sites where at least 1 unambiguous base is present in at least 1 sequence for each descendent clade is shown next to each internal node in the tree. This analysis involved 50 nucleotide sequences. There were a total of 685 positions in the final dataset. Evolutionary analyses were conducted in MEGA X^33^. The reliability of the tree was evaluated by the self-sampling method (bootstrap) with 2000 replications.

### Thiamine requirement

Thiamine requirement was tested for 8 isolates—HS10, HS23, HS27, HS29, HS30, HS38 and HS47. For this purpose, isolates were cultivated on the pure liquid Czapek-Dox medium (CzD) and liquid Czapek-Dox + thiamine medium (CzD-th) with a thiamine concentration of 10^–5^ M. The cultivation was carried out for 14 days at 23 ± 1 °C. The mycelium obtained at that time was filtered off, dried, and the dry mass was determined. The auxotrophy was determined according to the ratio of the dry mass of mycelium grown on a medium with the addition of thiamine (CzD-th) to the dry mass of mycelium grown on the medium without the addition of thiamine (CzD). Ratio CzD-th/CzD < 1.5 means prototrophic fungus, while ratio CzD-th/CzD > 1.5—the fungus is auxotrophic to thiamine^31^. The experiment was performed in four replications.

### Statistical analysis

Results were analyzed using ANOVA in Statistica software and Excel (*p* = 0.05). The significance of differences among results was calculated using the Duncan test (*p* = 0.05), and standard deviations were also calculated.

## Results

### Morphological identification, nuclear condition, and hyphae diameter

**Figure 2:**
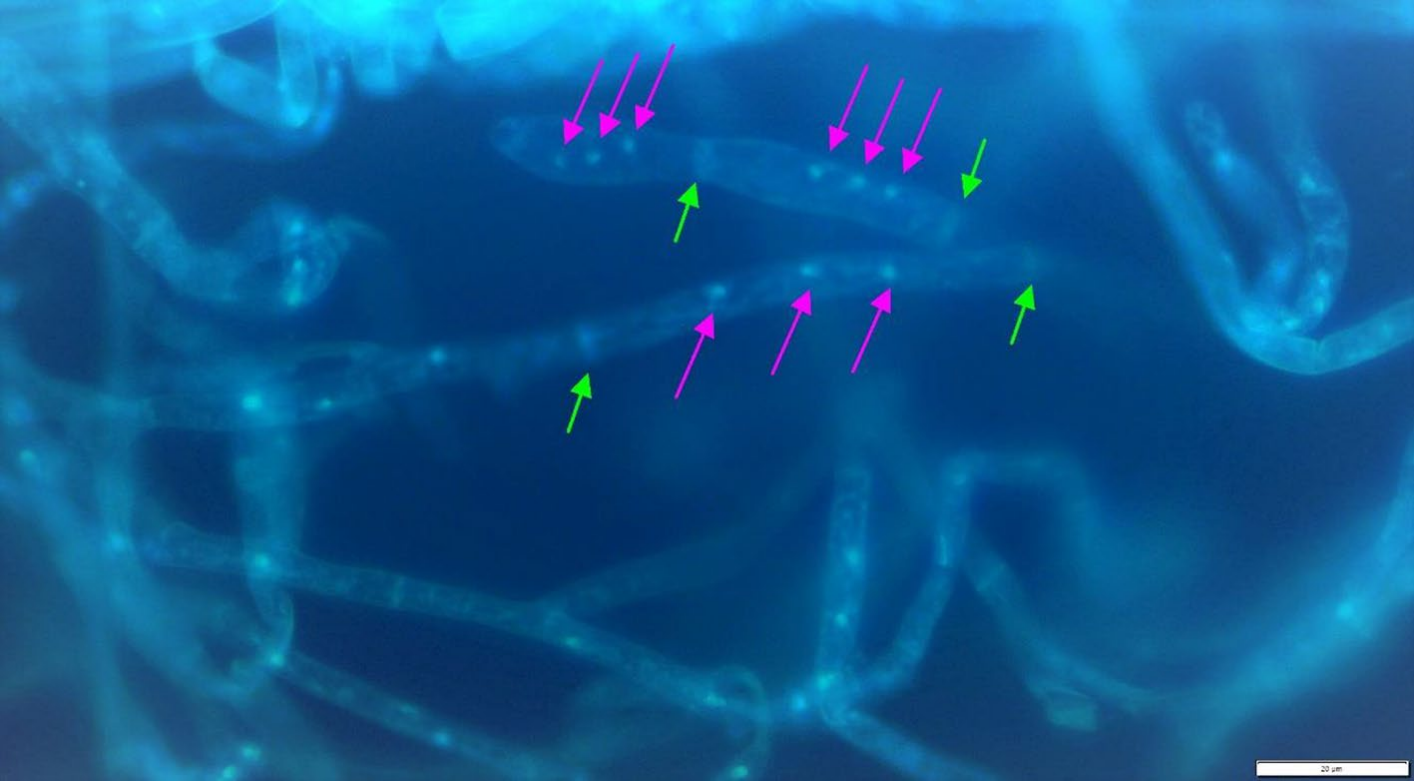
Nuclei condition in AG-E cells; pink arrows show nuclei, green arrows show cell walls (HS-30) (phot. E. Moliszewska).

Tested isolates were primarily accessed to the *Rhizoctonia* genus according to morphological features and after measurements of hyphae diameter, they were considered BNRs. The hyphae diameter ranged from 3.03 µm for HS21 to 5.27 µm for HS23 (Tab. 4). The average number of nuclei in cells of the test isolates ranged from 1.9 for the HS21 isolate to 3.6 for the HS10 and HS23 isolates (Tab. 4). There was a moderate positive correlation between the diameter of the hyphae and the number of nuclei in cells of tested isolates (correlation coefficient = 0,5088). Using this characteristic, the isolates were divided into two groups. In the first group were fungi which formed cells with only two nuclei (2n group), and in the second group, fungi which showed a varied number of nuclei, usually close to three (3n group) (Fig. 2, Table 4).

**Table 4:** The average number of nuclei per cell in tested binucleate *Rhizoctonia* isolates.

Isolate	Anastomosis group (classical method)	Anastomosis group (molecular method)	Average number of nuclei per cell	2n/3n group	Average hyphae diameter (μm)
HS21	–	AG-E	1,9	2n	3,03
HS27	AG-E	AG-E	2,1	2n	4,58
HS33	–	AG-E	2,3	2n	4,24
HS38	AG-E	AG-E	2,4	2n	4,78
HS29	AG-E	AG-E	2,5	3n	4,49
HS47	AG-E	AG-E	2,5	3n	5,17
HS46	AG-E	AG-E	2,7	3n	4,88
HS30	AG-E	AG-E	2,7	3n	4,68
HS45	-	AG-E	2,7	3n	4,58
HS26	-	AG-E	2,8	3n	4,53
HS10	-	AG-E	3,6	3n	4,59
HS23	-	AG-E	3,6	3n	5,27

AG-E isolates grown at room temperature (22 ± 1 °C) presented the majority of the fluffy, creamy, or brown mycelia; a few of them were white; with or without zonation on the culture surface. All of them developed monilioid cells and sclerotia, however, their abundance varied from poor ( +) for HS21, HS29, and HS26 to abundant (+ +  + +) for HS10, HS33, and HS47 (Table 5). There was no regularity and dependency in mycelium colour, its zonation and sclerotia presence according to the number of nuclei if they were considered as two groups (2-nucleic and 3-nucleic isolates).

**Table 5:** Morphological characteristics of binucleate *Rhizoctonia* isolates.

Isolate	AG-E subgroup	Aerial mycelium colour	Mycelium structure	Zonation	Monilioid cells	Sclerotia
HS21	2n	Creamy	Fluffy	+ *	+	+
HS27	2n	Brown	Fluffy	+	+	+ + +
HS33	2n	Brown	Fluffy	+	+	+ + + +
HS38	2n	Creamy	Fluffy, delicate	–	+	+ +
HS29	3n	Brown	Fluffy	+	+	+
HS47	3n	Creamy	Fluffy	–	+	+ + + +
HS46	3n	Creamy	Fluffy	+	+	+ + +
HS30	3n	Creamy	Fluffy	–	+	+ + +
HS45	3n	brown	Fluffy	–	+	+ +
HS26	3n	Creamy/ brown	Fluffy	+	+	+
HS10	3n	Brown	Profuse, fluffy	–	+	+ + + +
HS23	3n	Brown	Fluffy	+	+	+ + +

*Signs „ + ” or „-” mean presence or lack of the feature; „ + ”, „ +  + ”, „ +  +  + ” and „ +  +  +  + ” mean intensity of sclerotia creation where “ + ” means poor and “ +  +  +  + ” abundant.

### Molecular identification and phylogenetic analysis

**Figure 3:**
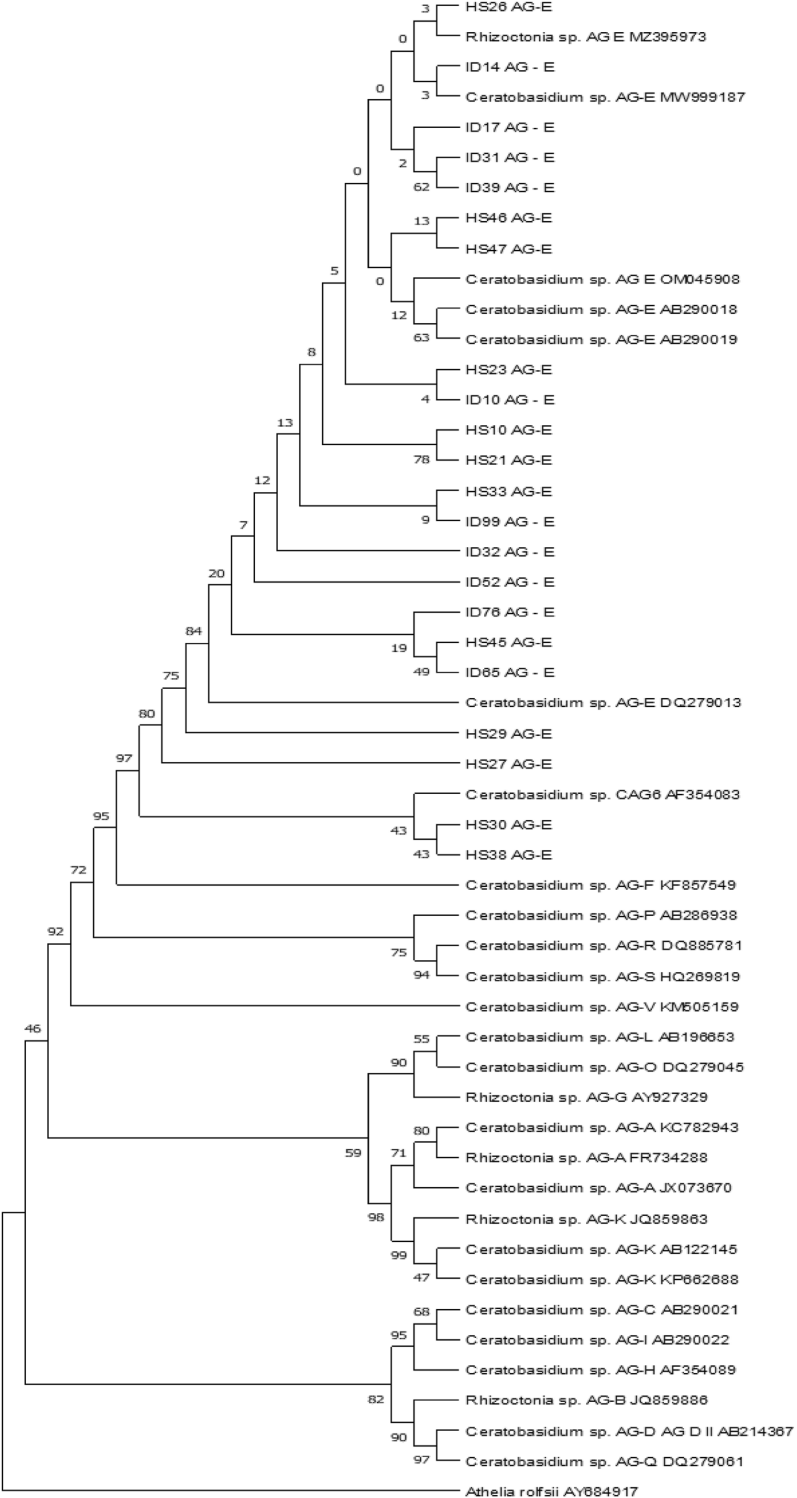
A bootstrap tree showing genetic relationships among the AG-E *Rhizoctonia* isolates based on the internal transcribed spacer sequences, the tree was rooted on the *Athelia rolfsii* (AY684917) sequence.

The molecular identification of tested isolates was based on the ITS1-ITS2 sequences and included a comparison to other isolates with the BLAST tool (“Supplementary Information”). This showed that our isolates belong to *Ceratobasidium* sp./*Rhizoctonia* sp. AG-E. Six of them and one CAG-6 were used as reference sequences in constructing a phylogenetic tree (Table 3, Fig. 3). The observed percentage identity in BLAST was over 99% with E value = 0 for most of our sequences; however, in some cases, e.g. HS29, it was lower, achieving the level 95% of identity to the sequence AB290018. The affiliation to AG-E was randomly confirmed for several isolates, listed in Table 4, by classical anastomosis fusion resulting in a C2 reaction (killing-reaction) when they were paired with tester AG-E isolates Lu-1 and RH155 and failing with other randomly used testers for other AGs (Table 2 and 4, Figs. 1 and 3).

### Phylogenetic relationships in BNR

Using phylogenetic analysis, a phylogenetic tree was composed of our isolates, both tested in this research and those listed in Table 7, they belong to both HS and ID groups. They were compared to reference isolates of AG-E and other AGs found in the GeneBank database. The ITS1-5.8S-ITS2 sequences of our isolates clustered with sequences of *Ceratobasidium* sp. AG-E isolates and one *Rhizoctonia* sp. AG-E isolate, apart from other anastomosis groups. Two sequences, HS30 and HS38, showed more comparability to a sequence of CAG-6 accession AF354083 (Fig. 2). There is no distinct separation of 2-nucleate and 3-nucleate isolates, which means that 3n isolates are not possible to separate by molecular methods based on ITS1-ITS2 sequence.

Searching accessions in GeneBank showed a broad spectrum of host plants from which isolates were obtained. Among various host plants, there were wheat, carrots, flax, bean, and *Erigeron* sp. representing weed plants, as opposed to crop plants (Table 4).

### Growth rates in different temperatures

**Figure 4:**
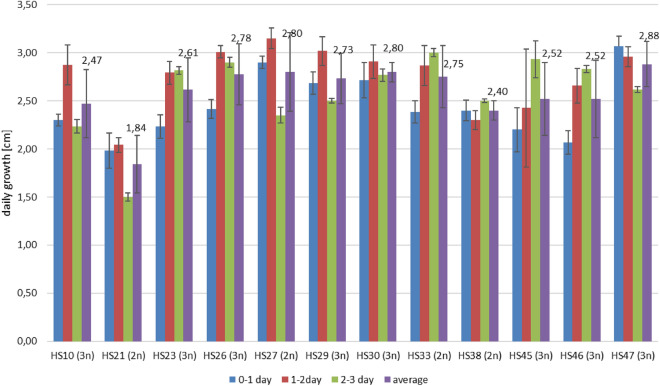
The daily rates of hyphal growth of binucleate *Rhizoctonia* isolates in 24 °C.

**Figure 5:**
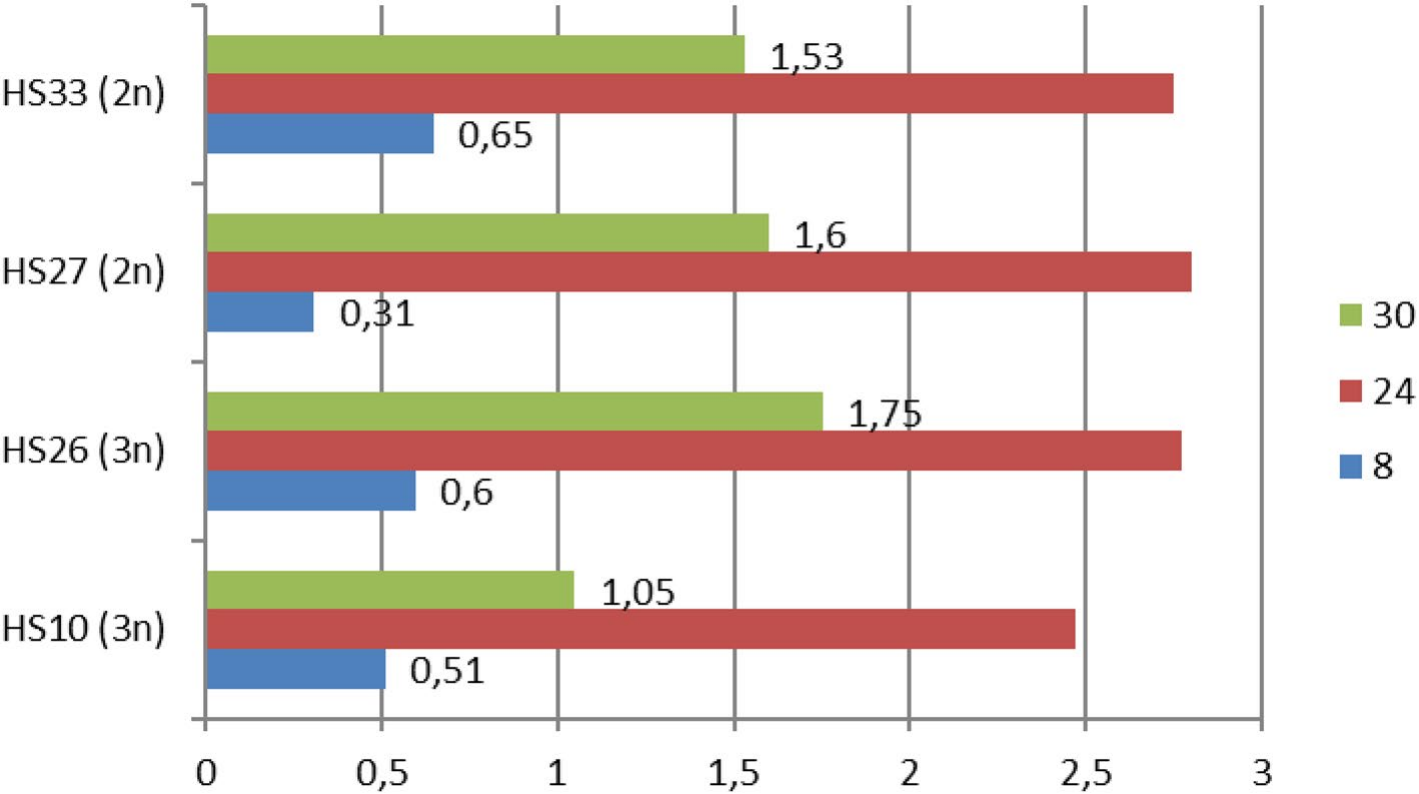
The daily rate of hyphal growth of selected binucleate *Rhizoctonia* isolates in 8, 24 and 30 °C.

The average growth rates of the tested BNR isolates ranged from 1.84 to 2.88 cm per day at 24 °C. The slowest growth was shown by the HS21 isolate, and the fastest by HS47 (Fig. 4). The statistical analyses did not allow separate homogeneous groups (According to Duncan’s test), which means that there were no significant differences among tested isolates in the average daily growth rate in 24 °C; however, the *p*-value for daily growth was at the level 0,001. Among them, 7 isolates presented the highest growth speed on the second day of growth, while 5 grew the fastest on the third day of development, and only one (HS47) grew the fastest on the first day of cultivation (Fig. 4). Lowering the temperature had a significant effect on the growth rate of the tested isolates. At 8 °C it was much slower than at 24 °C, but it was not completely inhibited—it ranged from 0.31 (HS27) to 0.65 (HS33) cm per day (Fig. 5). Increasing temperature to 30 °C caused increasing the growth rate, however, it was significantly inhibited comparing to the growth rates at 24 °C. The growth rates of BNR at 30 °C ranged from 1.05 cm/day (HS10 isolate) to 1.75 cm/day (HS26 isolate) (Fig. 5). Isolates differed in the growth rates at all tested temperatures, which was confirmed by Duncan’s test analysis. However, it was not possible to separate homogenous groups for the same temperature among tested strains, which shows that they belonged to only one group and behaved similarly.

### Thiamine requirement

The test for thiamine requirement in AG-E *Rhizoctonia* isolates showed that only two of the tested isolates were auxotrophic (HS29 and HS23) (Table 6). There is no possibility to determine any relationship between nuclei number and auxotrophy—this feature seems to exist only occasionally within this *Rhizoctonia* group.

**Table 6:** The ability of the tested binucleate *Rhizoctonia* isolates to synthesize thiamine (auxotrophic isolates are marked in bold).

Isolate	Anastomosis group	The average number of nuclei per cell	CzD dry mass [g]	CzD-th dry mass [g]	CzD-th/CzD
HS27	E	2,1/2n	0,27	0,18	0,67
HS38	E	2,4/2n	0,10	0,07	0,67
**HS29**	E	2,5/3n	0,30	0,62	**2,10**
HS47	E	2,5/3n	0,47	0,32	0,68
HS30	E	2,7/3n	0,18	0,23	1,24
HS10	E	3,6/3n	0,27	0,23	0,85
**HS23**	E	3,6/3n	0,13	0,27	**2,08**

## Discussion

The results of this research led to a description of a novel feature, based on an average nuclei number in cells, helpful in *Rhizoctonia* fungi determination. Additionally, tested isolates were described morphologically and microscopically. We did not find any atypical features in the morphology appearance of the group. They presented typical *Rhizoctonia* genus colors, creamy to brown, and hyphae structure, and they created monilioid cells and sclerotia, however with various abundance. The AG-E consists of both thiamine- auxotrophic and prototrophic isolates. The same was observed by Carling et al.^31^ in the case of *R. solani* AG-9, while for other anastomose groups, thiamine requirement is reported as characteristic for the whole group, thus thiamine requirement cannot be used as a diagnostic characteristic for AG-E isolates. The growth rates of tested isolates were quite fast at 24 °C, which seems to be typical for members of the *Rhizoctonia* genus, while it is slower at lower temperatures as well as at higher temperatures. Our isolates presented diverse sources of isolation, coming from herbaceous to woody plants.

Genus *Rhizoctonia* includes many species that are morphologically, ecologically, and genetically diverse. Their teleomorphs generally belong to the phylum Basidiomycota, and the class Agaricomycetes. Typically, the term “*Rhizoctonia*” is understood to mean fungi classified in the genus *Rhizoctonia* D.C. (syn. = *Moniliopsis* Ruhland.) with the teleomorph *Thanatephorus* Donk, for which the basionym is the multinucleate *R. solani* J.G. Kühn (family *Ceratobasidiaceae*). The binucleate “*Rhizoctonia*” (BNR) isolates with teleomorphic stage *Ceratobasidium* D.P. Rogers were formerly referred to as the taxon *Ceratorhiza* R.T. Moore. The type species is *C. goodyerae-repentis* (Constantin & L.M. Dufour) R.T. Moore, and its synonym is *Rhizoctonia goodyerae-repentis* Constantin & L.M. Dufour (family Ceratobasidiaceae). This large complex of fungi also includes genera that are often called “*Rhizoctonia*-like”: *Chrysorhiza, Thanatophytum, Opadorhiza, Epulorhiza,* and *Ascorhizoctonia* (family Corticiaceae, phylum Ascomycota)^6,35^. The informal basis for differentiation within *Rhizoctonia* spp. is the system of anastomosis groups. In the 1930s, Matsumoto et al. and Schultz, independently of each other, observed the phenomenon of anastomosis (fusion) between the hyphae of *Rhizoctonia* spp. isolates, and their research provided the basis for the currently used classification system for genus^5,36^. Anastomose groups are denoted by numbers for multinucleated *Rhizoctonia* spp. or by capital Latin letters for binucleate (BNR) strains. This system is informal, although widely recognized due to its usefulness in classifying and distinguishing individual isolates. Classification within the *Rhizoctonia* complex is based primarily on anamorphs since obtaining teleomorphs is difficult. Anastomosing reactions between *Rhizoctonia* spp. hyphae are related to the genetic relatedness of the isolates, but observing anastomose reactions is difficult, requires experience, and is time-consuming and laborious. This is why the comparison of sequences is a frequently chosen method for the determination of AG affiliation. Sharon et al.^11^ pointed out that the 95–100% sequence similarity and the localization of the ITS1-5.8S-ITS2 sequence on a phylogenetic tree within a cluster of a particular anastomosis group is sufficient to determine the identity of the studied isolate.

We confirmed the AG affiliation, primarily according to the sequence similarity, and then the anastomosis reaction gave us confirmation of our molecular findings. However, morphological observation showed us that on the microscopic level, our isolates differed in the number of nuclei per cell. Some of them distinctly showed three nuclei in the majority of cells. All of them belonged to AG-E. Simultaneously, within the AG-E, considered to be binucleate^13,35^, apart from the other binucleate AGs, we found isolates containing various numbers of nuclei in cells (from one to four), the average number of nuclei for some of them was about three, and many cells presented a distinctly tri-nucleic state. Moliszewska^20^ isolated several trinucleate isolates belonging to AG-E, indicating that research in this direction should be continued in order to determine whether this gives the basis for the separation of an individual subgroup of trinucleate *Rhizoctonia* spp. Furthermore, in the work of Adams and Butler^21^, there is a mention of the unusual trinucleate *Rhizoctonia* sp.; however, they did not define their affiliation to AG and did not undertake further considerations on this phenomenon. Our own observations regarding the number of nuclei in AG-E cells clearly indicate that there are both binucleate and trinucleate strains within this group, which can be the basis for dividing this group into two subgroups—binucleate (AG-E-2n) and trinucleate (AG-E-3n). It was also observed that the hyphae diameter of *Rhizoctonia* spp. isolates is related to the number of nuclei in the cell—larger hyphae diameters were usually found in trinucleate than in binucleate isolates, which was confirmed by the moderate positive correlation coefficient (0,5088) between these two features.

Sharon et al.^11^ indicated that the commonly accepted division into the bi- and multinucleate mycelia of *Rhizoctonia* spp. is not so simple, which is also confirmed by our own research. In Scandinavia, uninucleate isolates were found with the *Ceratobasidium bicorne* teleomorph^19,37,38^. Uninucleate *Rhizoctonia* spp. were also isolated by Otero et al.^2^ from tropical orchids in Central America and by Zhou et al.^39^ from grasses and maize in China. Hietala et al.^19^ and Otero et al.^2^ observed that some isolates formed both uni- and binucleate mycelium. In our collection, there were no uninucleate isolates. Additionally, it is worth mentioning that Ogoshi^4^ described four-nucleate isolates of *R. solani* AG-4 as an anamorph of *Tanatephorus praticola*, which is currently considered *R. solani*, however, Mordue et al.^40^ recognized definitely AG-4 as *T. praticola*, emphasizing it as separate species. Typically it is concluded that isolates containing four or more nuclei are considered *R. solani*, and those with fewer than four nuclei are concluded BNRs. The results of this research allow the conclusion that the determination of the average number of nuclei in *Rhizoctonia* spp. cells may be much more useful than previously thought. A typical diagnostic procedure for this type of fungi is microscopic observation (distinguishing characteristic hyphae with branching at almost a right angle and a septum located just behind the branching, lack of clump-connections), followed by measurement of the hyphae diameter and observation of the number of nuclei in the cells. This is the distinction between MNR and BNR. MNRs have wider hyphae (usually greater than 7 µm) and a large number of nuclei (4 to a dozen) in the individual cell. The last two steps are often omitted by researchers, resulting in misclassification. Our own observations suggest the necessity of counting nuclei in actively growing cells as routine management. If it is established that the mean number of nuclei is close to 3, it can be assumed that the isolate belongs to AG-E. Thus, in a simplified way, it is possible to determine the taxonomic affiliation of an isolate to AG-E. However, it should be remembered that within AG-E there are also binucleate isolates, which will not make it possible to negate the belonging to this group for binucleate mycelium and will be a reason for introducing other diagnostic techniques. Moliszewska^20^ analyzed several isolates of BNR and showed a 3-nuclei (3n) group of isolates giving some additional characteristics. She found that among 3n isolates, there are possibly two zymogram patterns, and simultaneously they also have similar ITS1-ITS2 sequences and were different from the AG-K isolates (Table 7). However, cluster grouping on the dendrogram does not support the observation of two zymogram patterns given by Moliszewska^20^ (Table 7).

**Table 7:** Characteristics of bi- and three-nucleate *Rhizoctonia* spp.; features which were of the same quality were assigned by one or two stars (*, **) (according to Moliszewska^20^; corrected).

Isolate	Anastomosis group	The average nuclei number	Qualification of the nuclei number	ITS1-ITS2 sequence group	Zymogram pattern	GeneBank Accession number
ID 31	E	3,01	3n	*	*	OQ646710
ID 32	E	3,05	3n	*	*	OQ646711
ID 52	E	3,00	3n	*	*	OQ646713
ID 76	E	3,09	3n	*	nd^a^	OQ646715
ID 99	E	3,00	3n	*	*	OQ646716
ID 39	E	2,88	3n	*	*	OQ646712
ID 65	E	2,97	3n	*	*	OQ646714
ID 10	E	2,60	3n	*	**	OQ646707
ID 14	E	2,87	3n	*	**	OQ646708
ID 17	E	2,97	3n	*	**	OQ646709
ID 57	K	2,02	2n	**	***	OQ646866
ID 58	K	2,05	2n	**	***	OQ646867
ID 68	K	2,00	2n	**	***	OQ646869
ID 73	K	2,35	2n	**	***	OQ646870
ID 60	K	2,15	2n	**	***	OQ646868

^a^*nd* not determined: asterisks (*, **, ***) are used to determine the same pattern of sequence or zymogram.

Rovira et al.^41^ reported that the ability to synthesize thiamine is a property of the whole anastomose group, not an individual strain. Despite this, fungi of the genus *Rhizoctonia* may be divided into groups or subgroups based on the ability to synthesize thiamine, but we did not observe such results among AG-E isolates. There were thiamine auxotrophs and autotrophs within this group; however, thiamine auxotrophy seems less frequent than thiamine autotrophy.

Isolates of *Rhizoctonia* sp. AG-E are able to infect various plant species, including crop plants as well as trees and weeds. Our isolates came partly from trees, like scot pine and silver birch, and partly from crop plants such as lupin, white mustard, vetch, and sugar beets. AG-E testers were isolated from cocksfoot and flax, and searching the GeneBank database brought several other host-plant species such as wheat, carrots, flax, bean, and *Erigeron* sp. (Tables 1, 2, 3). Those plants belong to various taxonomic groups, thus the AG-E isolates do not show any specific specialty, or on the contrary, they present a very broad specialty against host plants.

## Conclusions

The current statement of nuclear conditions possible to observe in the *Rhizoctonia* genus indicates five possible average numbers of nuclei in cells, namely strains that may be uni-nucleate, binucleate, three-nucleate, four-nucleate, and multiple-nucleate. According to our findings, we propose a simplification in the method for diagnosis of trinucleate strains of the *Rhizoctonia*-like group. Directly observing the tri-nucleate (3n) condition allows strains to be identified as *Rhizoctonia* sp. AG-E; however, we strongly suggest also using the sequencing method for confirmation of the AG statement.

### Supplementary Information

Supplementary Information.

## Data Availability

The DNA sequences generated and analyzed during the current study are available in the GeneBank (NCBI) database under the accession numbers: KX831947, KX831953, KX831955, KX831957–KX831961, KX831963, KX831965–KX831967, OQ646707–OQ646716, OQ646866–OQ646870.

## References

[1] Kuramae EE, Buzeto AL, Nakatani AK, Souza NL (2007). rDNA based characterization of a new binucleate *Rhizoctonia* spp. causing root rot on kale in Brasil. Eur. J. Plant Pathol..

[2] Otero JT, Ackerman JD, Bayman P (2002). Diversity and host specificity of endophytic *Rhizoctonia*-like fungi from tropical orchids. Am. J. Bot..

[3] Maculewicz D (2015). Binucleate *Rhizoctonia* spp. as biocontrol agents against plant pathogens. Ecol. Chem. Eng. A.

[4] Ogoshi A, Sneh B, Jabaji-Hare S, Neate S, Dijst G (1996). Introduction—the genus *Rhizoctonia*. Rhizoctonia Species: Taxonomy, Molecular Biology, Ecology, Pathology and Disease Control.

[5] Sneh B, Burpee L, Ogoshi A (1991). Identification of Rhizoctonia Species.

[6] Moliszewska E, Maculewicz D (2016). Dwujądrowe *Rhizoctonia* spp. jako patogeny roślin. Eduk. Biol. Środowiskowa.

[7] Ogoshi A, Ui T (1979). Specificity in vitamin requirement among anastomosis groups of *Rhizoctonia solani* Kuhn. Ann. Phytopath. Soc. Japan.

[8] Priyatmojo A (2001). Characterization of a new subgroup of *Rhizoctonia solani* anastomosis group 1 (AG-1-ID), causal agent of a necrotic leaf spot on coffee. Phytopathology.

[9] Sharon M, Freeman S, Kuninaga S, Sneh B (2007). Genetic diversity, anastomosis groups, and virulence of *Rhizoctonia* spp. from strawberry. Eur. J. Plant Pathol..

[10] Yang YG, Zhao C, Guo ZJ, Wu XH (2015). Characterization of a new anastomosis group (AG-W) of binucleate *Rhizoctonia*, causal agent of potato stem canker. Plant Dis..

[11] Sharon M, Kuninaga S, Hyakumachi M, Naito S, Sneh B (2008). Classification of *Rhizoctonia* spp. using rDNA-ITS sequence analysis supports the genetic basis of the classical anastomosis grouping. Mycoscience.

[12] Dong W (2017). Identification of AG-V, a new anastomosis group of binucleate *Rhizoctonia* spp. from taro and ginger in Yunnan province. Eur. J Plant Pathol..

[13] Ogoshi A, Oniki M, Araki T, Ui T (1983). Studies on the anastomosis groups of binucleate *Rhizoctonia* and their perfect states. J. Fac. Agric. Hokkaido Univ..

[14] Ogoshi A, Oniki M, Araki T, Ui T (1983). Anastomosis groups of binucleate *Rhizoctonia* in Japan and in North America and their perfect states. Trans. Mycol. Soc. Jpn..

[15] Burpee LL, Sanders PL, Cole H, Sherwood RT (1980). Anastomosis groups among isolates of *Ceratobasidium cornigerum* and related fungi. Mycologia.

[16] Hyakumachi M, Priyatmojo A, Kubota M, Fukui H (2005). New anastomosis groups, AG-T and AG-U, of binucleate *Rhizoctonia* spp. causing root and stem rot of cut-flower and miniature roses. Phytopathology.

[17] Kuninaga S (2002). Current situation of the taxonomy of the genus *Rhizoctonia* and the *R. solani* species complex. Jpn. J. Phytopathol..

[18] Gonzalez D, Carling DE, Kuninaga S, Vilgalys R, Cubeta MA (2001). Ribosomal DNA systematics of *Ceratobasidium* and *Thanatephorus* with *Rhizoctonia* anamorphs. Mycologia.

[19] Hietala AM, Sen R, Lilja A (1994). Anamorphic and teleomorphic characteristics of a uninucleate *Rhizoctonia* spp. isolated from the roots of nursery grown conifer seedlings. Mycol. Res..

[20] Moliszewska, E. B. Etiologia wybranych chorób buraka cukrowego (ed. Wydawnictwo Uniwersytetu Opolskiego) 85–89 (in Polish) (Opole 2009).

[21] Adams GC, Butler EE (1983). Influence of nutrition on the formation of basidia and basidiospores in *Thanatephorus cucumeris*. Phytopathology.

[22] Sneh B, Adams GC, Sneh B, Jabaji-Hare S, Neate S, Dijst G (1996). Culture preservation methods for maintaining genetic integrity of Rhizoctonia spp. isolates. Rhizoctonia Species: Taxonomy, Molecular Biology, Ecology, Pathology and Disease Control.

[23] Bandoni RJ (1979). Safranin O as a rapid nuclear stain for fungi. Mycologia.

[24] Murray MG, Thompson WF (1980). Rapid isolation of high molecular weight plant DNA. Nucleic Acid Res..

[25] Wagner DB (1987). Chloroplast DNA polymorphisms in lodgepole and jack pines and their hybrids. Proc. Nat. Acad. Sci. USA.

[26] White TJ, Bruns T, Lee S, Taylor J, Innis MA, Gelfand DH, Sninsky JJ, White TJ (1990). Amplification and direct sequencing of fungal ribosomal RNA genes for phylogenetics. PCR Protocols: A Guide to Methods and Applications.

[27] He F (2011). Standard PCR protocol. Bio-protocol.

[28] Altschul SF, Gish W, Miller W, Myers EW, Lipman DJ (1990). Basic Local Alignment Search Tool. J. Mol Biol..

[29] Kronland WC, Stanghellini ME (1988). Clean slide technique for the observation of anastomosis and nuclear condition of *Rhizoctonia solani*. Phytopathology.

[30] Moliszewska EB, Schneider JHM (2002). Some pathogenic properties of *Rhizoctonia solani* to sugar beet seedlings. Plant Protect. Sci..

[31] Carling DE, Leiner RH, Kebler KM (1987). Characterization of a new anastomosis group (AG-9) of *Rhizoctonia solani*. Phytopathology.

[32] Thompson JD, Higgins DG, Gibson TJ (1994). CLUSTAL W: Improving the sensitivity of progressive multiple sequence alignment through sequence weighting, position-specific gap penalties and weight matrix choice. Nucleic Acids Res..

[33] Kumar S, Stecher G, Li M, Knyaz C, Tamura K (2018). MEGA X: Molecular evolutionary genetics analysis across computing platforms. Mol. Biol Evol..

[34] Tamura K, Nei M (1993). Estimation of the number of nucleotide substitutions in the control region of mitochondrial DNA in humans and chimpanzees. Mol. Biol. Evol..

[35] Gonzalez Garcia V, Portal Onco MA, Rubio Susan V (2006). Review. Biology and systematics of the form genus *Rhizoctonia*. Span. J. Agric. Res..

[36] Matsumoto T, Yamamoto W, Hirane S (1932). Physiology and parasitology of the fungi generally referred to as *Hypochnus sasakii* Shirai I. Differentiation of the strains by means of hyphal fusion and culture in differential media. J. Soc. Trop. Agric..

[37] Lilja A, Hietala AM, Karjalainen R (1996). Identification of a uninucleate *Rhizoctonia* sp. by pathogenicity, hyphal anastomosis and RAPD analysis. Plant Pathol..

[38] Hietala AM, Vahala J, Hantula J (2001). Molecular evidence suggests that *Ceratobasidium bicorne* has an anamorph known as a conifer pathogen. Mycol. Res..

[39] Zhou S (2015). A uninucleate *Rhizoctonia* sp. from maize plant with ITS heterogeneity and hypersensitive to abiotic stresses. Eur. J. Plant. Pathol..

[40] Mordue JEM, Currah RS, Bridge PD (1989). An integrated approach to *Rhizoctonia* taxonomy: Cultural, biochemical and numerical techniques. Mycol. Res..

[41] Rovira AD, Ogoshi A, MacDonald HJ (1986). Characterization of isolates of *Rhizoctonia solani* from cereal roots in South Australia and New South Wales. Phytopathology.

