# Supplementary material for: Characterization of three-nucleate Rhizoctonia AG-E based on their morphology and phylogeny
Source: Sci Rep. 2023 Oct 13;13:17328. doi: 10.1038/s41598-023-44448-1 (PMC10575891; doi:10.1038/s41598-023-44448-1)
Supplement: Supplementary file 1 — Supplementary Information. [file 41598_2023_44448_MOESM1_ESM.docx]

Table A- Isolates identity [%]

| Nr | Isolate code | 1 | 2 | 3 | 4 | 5 | 6 | 7 | 8 | 9 | 10 | 11 | 12 | 13 | 14 | 15 | 16 | 17 | 18 | 19 | 20 | 21 | 22 | 23 | 24 | 25 | 26 | 27 | 28 | 29 | 30 | 31 | 32 | 33 | 34 | 35 | 36 | 37 | 38 | 39 | 40 | 41 | 42 | 43 | 44 | 45 | 46 |
| --- | --- | --- | --- | --- | --- | --- | --- | --- | --- | --- | --- | --- | --- | --- | --- | --- | --- | --- | --- | --- | --- | --- | --- | --- | --- | --- | --- | --- | --- | --- | --- | --- | --- | --- | --- | --- | --- | --- | --- | --- | --- | --- | --- | --- | --- | --- | --- |
| 1 | ID10 | 100 |  |  |  |  |  |  |  |  |  |  |  |  |  |  |  |  |  |  |  |  |  |  |  |  |  |  |  |  |  |  |  |  |  |  |  |  |  |  |  |  |  |  |  |  |  |
| 2 | ID14 | 99,85 | 100 |  |  |  |  |  |  |  |  |  |  |  |  |  |  |  |  |  |  |  |  |  |  |  |  |  |  |  |  |  |  |  |  |  |  |  |  |  |  |  |  |  |  |  |  |
| 3 | ID17 | 99,85 | 99,71 | 100 |  |  |  |  |  |  |  |  |  |  |  |  |  |  |  |  |  |  |  |  |  |  |  |  |  |  |  |  |  |  |  |  |  |  |  |  |  |  |  |  |  |  |  |
| 4 | ID31 | 99,85 | 99,71 | 99,71 | 100 |  |  |  |  |  |  |  |  |  |  |  |  |  |  |  |  |  |  |  |  |  |  |  |  |  |  |  |  |  |  |  |  |  |  |  |  |  |  |  |  |  |  |
| 5 | ID32 | 98,98 | 98,83 | 98,84 | 98,83 | 100 |  |  |  |  |  |  |  |  |  |  |  |  |  |  |  |  |  |  |  |  |  |  |  |  |  |  |  |  |  |  |  |  |  |  |  |  |  |  |  |  |  |
| 6 | ID39 | 99,85 | 99,71 | 99,71 | 99,85 | 98,83 | 100 |  |  |  |  |  |  |  |  |  |  |  |  |  |  |  |  |  |  |  |  |  |  |  |  |  |  |  |  |  |  |  |  |  |  |  |  |  |  |  |  |
| 7 | ID52 | 99,67 | 99,5 | 99,7 | 99,5 | 99 | 99,5 | 100 |  |  |  |  |  |  |  |  |  |  |  |  |  |  |  |  |  |  |  |  |  |  |  |  |  |  |  |  |  |  |  |  |  |  |  |  |  |  |  |
| 8 | ID65 | 100 | 99,83 | 100 | 99,83 | 99,83 | 99,83 | 99,81 | 100 |  |  |  |  |  |  |  |  |  |  |  |  |  |  |  |  |  |  |  |  |  |  |  |  |  |  |  |  |  |  |  |  |  |  |  |  |  |  |
| 9 | ID76 | 100 | 99,81 | 100 | 99,81 | 94,78 | 99,81 | 99,81 | 100 | 100 |  |  |  |  |  |  |  |  |  |  |  |  |  |  |  |  |  |  |  |  |  |  |  |  |  |  |  |  |  |  |  |  |  |  |  |  |  |
| 10 | ID99 | 99,85 | 99,71 | 99,71 | 99,71 | 98,98 | 99,71 | 99,83 | 100 | 100 | 100 |  |  |  |  |  |  |  |  |  |  |  |  |  |  |  |  |  |  |  |  |  |  |  |  |  |  |  |  |  |  |  |  |  |  |  |  |
| 11 | HS10 | 99,38 | 99,22 | 99,38 | 99,23 | 98,45 | 99,22 | 99,33 | 99,65 | 99,62 | 99,53 | 100 |  |  |  |  |  |  |  |  |  |  |  |  |  |  |  |  |  |  |  |  |  |  |  |  |  |  |  |  |  |  |  |  |  |  |  |
| 12 | HS21 | 99,38 | 99,22 | 99,38 | 99,22 | 98,45 | 99,22 | 99,5 | 99,44 | 99,62 | 99,53 | 99,07 | 100 |  |  |  |  |  |  |  |  |  |  |  |  |  |  |  |  |  |  |  |  |  |  |  |  |  |  |  |  |  |  |  |  |  |  |
| 13 | HS23 | 100 | 99,84 | 100 | 99,85 | 99 | 99,84 | 99,67 | 100 | 100 | 99,84 | 99,06 | 99,53 | 100 |  |  |  |  |  |  |  |  |  |  |  |  |  |  |  |  |  |  |  |  |  |  |  |  |  |  |  |  |  |  |  |  |  |
| 14 | HS26 | 100 | 99,84 | 100 | 99,85 | 98,91 | 99,84 | 99,67 | 100 | 100 | 99,84 | 99,23 | 99,38 | 100 | 100 |  |  |  |  |  |  |  |  |  |  |  |  |  |  |  |  |  |  |  |  |  |  |  |  |  |  |  |  |  |  |  |  |
| 15 | HS27 | 96,12 | 95,97 | 96,12 | 96 | 95,2 | 96 | 96,36 | 97,02 | 97,35 | 96,27 | 95,8 | 95,59 | 96,12 | 96,11 | 100 |  |  |  |  |  |  |  |  |  |  |  |  |  |  |  |  |  |  |  |  |  |  |  |  |  |  |  |  |  |  |  |
| 16 | HS29 | 95,75 | 95,91 | 95,75 | 95,59 | 94,78 | 95,59 | 95,87 | 96,84 | 97 | 95,91 | 95,29 | 95,58 | 95,75 | 95,74 | 95,72 | 100 |  |  |  |  |  |  |  |  |  |  |  |  |  |  |  |  |  |  |  |  |  |  |  |  |  |  |  |  |  |  |
| 17 | HS30 | 96,8 | 96,93 | 96,8 | 96,63 | 95,9 | 96,63 | 97,04 | 97,23 | 97,57 | 96,93 | 96,45 | 96,64 | 96,77 | 96,76 | 95,69 | 96,53 | 100 |  |  |  |  |  |  |  |  |  |  |  |  |  |  |  |  |  |  |  |  |  |  |  |  |  |  |  |  |  |
| 18 | HS33 | 99,54 | 99,39 | 99,54 | 99,39 | 98,62 | 99,39 | 99,83 | 99,83 | 100 | 99,69 | 99,38 | 99,69 | 99,84 | 99,69 | 96,13 | 95,92 | 97,07 | 100 |  |  |  |  |  |  |  |  |  |  |  |  |  |  |  |  |  |  |  |  |  |  |  |  |  |  |  |  |
| 19 | HS38 | 98 | 98,15 | 98 | 97,85 | 97,1 | 97,85 | 98,02 | 98,45 | 98,5 | 98,15 | 97,41 | 98,01 | 98,13 | 97,99 | 96,32 | 97,05 | 98,62 | 98,31 | 100 |  |  |  |  |  |  |  |  |  |  |  |  |  |  |  |  |  |  |  |  |  |  |  |  |  |  |  |
| 20 | HS45 | 99,69 | 99,54 | 99,69 | 99,54 | 98,61 | 99,54 | 99,34 | 100 | 100 | 99,54 | 99,38 | 99,07 | 99,69 | 99,84 | 95,69 | 95,29 | 96,46 | 99,38 | 97,7 | 100 |  |  |  |  |  |  |  |  |  |  |  |  |  |  |  |  |  |  |  |  |  |  |  |  |  |  |
| 21 | HS46 | 99,37 | 99,21 | 99,37 | 99,21 | 98,43 | 99,21 | 99,34 | 99,47 | 99,44 | 99,53 | 99,05 | 99,07 | 99,07 | 99,37 | 95,96 | 95,42 | 96,71 | 99,53 | 97,66 | 98,91 | 100 |  |  |  |  |  |  |  |  |  |  |  |  |  |  |  |  |  |  |  |  |  |  |  |  |  |
| 22 | HS47 | 99,53 | 99,37 | 99,53 | 99,37 | 98,43 | 99,37 | 99,17 | 99,47 | 99,44 | 99,37 | 98,9 | 98,33 | 99,39 | 99,53 | 95,12 | 95,26 | 95,9 | 98,77 | 97,11 | 99,21 | 98,46 | 100 |  |  |  |  |  |  |  |  |  |  |  |  |  |  |  |  |  |  |  |  |  |  |  |  |
| 23 | AG-E MW999187 | 100 | 100 | 99,85 | 99,85 | 99 | 99,85 | 99,67 | 100 | 100 | 99,85 | 99,37 | 99,53 | 100 | 100 | 96,23 | 95,75 | 96,88 | 99,69 | 98,12 | 99,68 | 99,37 | 99,53 | 100 |  |  |  |  |  |  |  |  |  |  |  |  |  |  |  |  |  |  |  |  |  |  |  |
| 24 | AG-E OM045908 | 99,85 | 99,85 | 99,7 | 99,7 | 99 | 99,7 | 99,5 | 99,82 | 99,81 | 99,7 | 99,21 | 99,37 | 99,84 | 99,84 | 96,08 | 95,76 | 97,05 | 95,76 | 98,28 | 99,53 | 99,21 | 99,37 | 99,85 | 100 |  |  |  |  |  |  |  |  |  |  |  |  |  |  |  |  |  |  |  |  |  |  |
| 25 | AG-E MZ395973 | 100 | 100 | 99,85 | 99,85 | 99 | 99,85 | 99,67 | 100 | 100 | 99,85 | 99,36 | 99,52 | 100 | 100 | 96,18 | 95,75 | 96,85 | 95,75 | 98,1 | 99,68 | 99,36 | 99,52 | 100 | 99,85 | 100 |  |  |  |  |  |  |  |  |  |  |  |  |  |  |  |  |  |  |  |  |  |
| 26 | AG-E DQ279013 | 99,53 | 99,52 | 99,52 | 99,36 | 98,4 | 99,36 | 99,17 | 99,46 | 99,44 | 99,36 | 98,88 | 99,04 | 99,52 | 99,52 | 96,03 | 95,6 | 96,69 | 95,6 | 97,94 | 99,2 | 98,89 | 99,04 | 99,52 | 99,36 | 99,52 | 100 |  |  |  |  |  |  |  |  |  |  |  |  |  |  |  |  |  |  |  |  |
| 27 | AG-E AB290019 | 99,53 | 99,53 | 99,53 | 99,38 | 98,62 | 99,38 | 99,17 | 99,46 | 99,44 | 99,53 | 98,88 | 99,04 | 99,52 | 99,52 | 95,71 | 95,43 | 96,69 | 95,43 | 97,94 | 99,2 | 98,89 | 99,04 | 99,53 | 99,69 | 99,53 | 99,05 | 100 |  |  |  |  |  |  |  |  |  |  |  |  |  |  |  |  |  |  |  |
| 28 | AG-E AB290018 | 99,38 | 99,38 | 99,38 | 99,22 | 98,44 | 99,22 | 99,33 | 99,46 | 99,44 | 99,38 | 99,04 | 99,2 | 99,36 | 99,36 | 95,86 | 95,42 | 96,52 | 95,42 | 97,77 | 99,04 | 99,04 | 98,88 | 97,68 | 99,22 | 99,38 | 98,89 | 99,07 | 100 |  |  |  |  |  |  |  |  |  |  |  |  |  |  |  |  |  |  |
| 29 | CAG6 AF354083 | 97,72 | 97,72 | 97,72 | 97,57 | 97,22 | 97,57 | 97,52 | 98,1 | 97,94 | 97,87 | 97,38 | 97,37 | 97,65 | 97,68 | 95,73 | 96,89 | 98,15 | 97,55 | 99,39 | 97,39 | 97,33 | 97,18 | 99,38 | 97,84 | 97,65 | 97,47 | 97,5 | 97,34 | 100 |  |  |  |  |  |  |  |  |  |  |  |  |  |  |  |  |  |
| 30 | AG-F KF857549 | 94,79 | 94,79 | 94,65 | 94,65 | 94,37 | 94,65 | 94,23 | 95,37 | 94,98 | 94,93 | 94,31 | 94,3 | 94,53 | 94,61 | 92,89 | 93,15 | 93,74 | 94,5 | 94,95 | 94,33 | 94,37 | 94,06 | 94,69 | 94,53 | 93,63 | 94,64 | 93,99 | 94,13 | 94,86 | 100 |  |  |  |  |  |  |  |  |  |  |  |  |  |  |  |  |
| 31 | AG-P AB286938 | 91,79 | 91,77 | 91,77 | 91,62 | 90,71 | 91,62 | 93,73 | 93,94 | 93,66 | 91,91 | 93,81 | 93,81 | 94 | 94 | 92,1 | 92,01 | 92,81 | 94,16 | 93,81 | 94 | 93,84 | 93,48 | 91,77 | 91,63 | 91,77 | 94,18 | 91,48 | 91,6 | 93,47 | 91,31 | 100 |  |  |  |  |  |  |  |  |  |  |  |  |  |  |  |
| 32 | AG-S HQ269819 | 91,64 | 91,64 | 91,51 | 91,51 | 90,77 | 91,5 | 92,13 | 93,41 | 92,86 | 91,78 | 92,53 | 92,35 | 92,56 | 92,7 | 92,27 | 89,78 | 90,73 | 92,71 | 93,2 | 92,71 | 92,4 | 92,07 | 91,48 | 91,36 | 91,39 | 92,64 | 90,64 | 90,92 | 91,72 | 92,36 | 92,17 | 100 |  |  |  |  |  |  |  |  |  |  |  |  |  |  |
| 33 | AG-V KM505159 | 91,61 | 91,61 | 91,48 | 91,48 | 90,84 | 91,48 | 90,61 | 95,62 | 95,41 | 91,74 | 90,8 | 90,91 | 91,09 | 91,07 | 89,82 | 89,41 | 90,53 | 91,15 | 91,58 | 90,83 | 91,23 | 90,63 | 91,58 | 91,46 | 91,5 | 91,16 | 90,76 | 91,03 | 98,14 | 92 | 89,52 | 90,79 | 100 |  |  |  |  |  |  |  |  |  |  |  |  |  |
| 34 | AG-R DQ885781 | 88,98 | 88,98 | 99,98 | 88,84 | 88,11 | 88,84 | 89,51 | 90,32 | 89,7 | 89,11 | 89,95 | 89,89 | 90,06 | 90,09 | 89,1 | 88,57 | 88,53 | 90,27 | 90,75 | 90,13 | 89,9 | 89,74 | 85,26 | 88,79 | 88,79 | 89,95 | 88,3 | 88,56 | 89,26 | 90,2 | 89,88 | 92,2 | 88,71 | 100 |  |  |  |  |  |  |  |  |  |  |  |  |
| 35 | AG-O DQ279045 | 88,31 | 88,31 | 88,31 | 88,14 | 87,76 | 88,14 | 87,79 | 88,1 | 87,5 | 88,46 | 88,12 | 88,12 | 88,31 | 88,31 | 86,76 | 86,84 | 87,58 | 88,46 | 88,72 | 88,31 | 88,14 | 87,8 | 88,31 | 88,16 | 88,31 | 88,49 | 87,8 | 88,12 | 88,38 | 88,22 | 90,16 | 89,98 | 92,7 | 86,05 | 100 |  |  |  |  |  |  |  |  |  |  |  |
| 36 | AG-G AY927329 | 86,77 | 86,77 | 86,77 | 86,62 | 85,76 | 86,62 | 87,26 | 87,7 | 87,13 | 86,9 | 87,61 | 87,61 | 87,8 | 87,8 | 86,08 | 86,34 | 87,08 | 87,95 | 88,05 | 87,8 | 87,63 | 87,29 | 86,77 | 86,64 | 86,77 | 87,99 | 86,38 | 86,51 | 87,71 | 86,02 | 88,93 | 88,89 | 86,3 | 87,48 | 97,33 | 100 |  |  |  |  |  |  |  |  |  |  |
| 37 | AG-A KC782943 | 85,71 | 85,71 | 85,59 | 85,57 | 84,3 | 85,57 | 85,97 | 89,27 | 88,45 | 85,84 | 86,69 | 86,77 | 86,73 | 86,83 | 87,86 | 88,8 | 86,18 | 87,18 | 87,56 | 86,87 | 86,4 | 88,74 | 85,84 | 85,84 | 85,92 | 86,58 | 86,24 | 86,2 | 87,08 | 85,67 | 88,32 | 87,52 | 85,16 | 87,88 | 91,69 | 91,2 | 100 |  |  |  |  |  |  |  |  |  |
| 38 | AG-K AB122145 | 85,26 | 85,26 | 85,26 | 85,11 | 89,06 | 85,11 | 85,77 | 89,28 | 88,45 | 85,25 | 86,17 | 86,17 | 86,5 | 86,5 | 87,5 | 88,66 | 88,48 | 86,5 | 86,57 | 86,5 | 86,33 | 89,28 | 85,26 | 85,41 | 85,26 | 86,36 | 85,02 | 84,71 | 86,24 | 84,65 | 87,95 | 85,91 | 90,3 | 89,03 | 90,3 | 89,8 | 95,28 | 100 |  |  |  |  |  |  |  |  |
| 39 | AG-K JQ859863 | 84,74 | 84,74 | 84,62 | 84,59 | 83,63 | 84,59 | 85,85 | 88,56 | 88,73 | 84,74 | 85,65 | 85,44 | 85,69 | 85,95 | 86,55 | 87,27 | 87,38 | 85,83 | 85,76 | 85,97 | 88,29 | 88,29 | 84,45 | 84,49 | 84,29 | 85,36 | 85,19 | 85 | 85,58 | 84,47 | 86,46 | 83,59 | 83,61 | 87,48 | 90,24 | 88,2 | 93,54 | 97,35 | 100 |  |  |  |  |  |  |  |
| 40 | AG-B JQ859886 | 84,77 | 84,77 | 84,65 | 84,63 | 83,25 | 84,63 | 84,08 | 88,29 | 87,15 | 84,89 | 85,05 | 84,84 | 84,94 | 85,21 | 87,01 | 86,44 | 84,43 | 85,23 | 85,48 | 85,23 | 84,73 | 84,78 | 84,48 | 84,38 | 84,32 | 84,6 | 83,46 | 83,56 | 85,29 | 85,24 | 86,64 | 84,36 | 85,53 | 84,54 | 87,32 | 87,28 | 87,92 | 86,64 | 86,33 | 100 |  |  |  |  |  |  |
| 41 | AG-L AB196653 | 86,5 | 86,5 | 86,5 | 86,32 | 86,37 | 86,32 | 86,5 | 86,2 | 86,19 | 86,66 | 86,3 | 86,3 | 86,5 | 86,5 | 84,88 | 85,59 | 86,17 | 86,66 | 87,01 | 86,5 | 86,32 | 86,97 | 86,5 | 86,35 | 86,5 | 86,7 | 86,15 | 86,3 | 86,65 | 86,48 | 88,27 | 88,21 | 90,06 | 87,2 | 96,62 | 95,58 | 91,54 | 90,09 | 90,15 | 87,23 | 100 |  |  |  |  |  |
| 42 | AG-C AB290021 | 83,94 | 83,94 | 83,94 | 83,94 | 82,79 | 83,94 | 84,89 | 85,11 | 84,42 | 84,07 | 85,16 | 85,16 | 85,35 | 85,35 | 83,17 | 83,62 | 84,34 | 85,5 | 85,33 | 85,35 | 85,16 | 84,85 | 83,94 | 83,82 | 83,94 | 85,38 | 85,02 | 85,47 | 85,16 | 85,23 | 86,13 | 85,08 | 87,95 | 85,8 | 88,25 | 87,91 | 86,75 | 85,54 | 84,1 | 86,75 | 86,85 | 100 |  |  |  |  |
| 43 | AG-I AB290022 | 84,05 | 84,05 | 84,05 | 84,05 | 89,25 | 84,05 | 84,81 | 89,44 | 88,84 | 84,18 | 85,25 | 85,25 | 85,45 | 85,45 | 88,34 | 88,74 | 84,65 | 85,6 | 85,76 | 85,45 | 85,25 | 84,94 | 84,05 | 83,92 | 84,05 | 85,47 | 85,28 | 85,57 | 85,42 | 85,49 | 86,06 | 85,52 | 90 | 87,12 | 88,41 | 88,08 | 86,98 | 86,07 | 90,95 | 87,42 | 87,07 | 94,23 | 100 |  |  |  |
| 44 | AG-H AF354089 | 85,25 | 85,25 | 85,25 | 85,08 | 88,92 | 85,08 | 84,25 | 89,05 | 88,16 | 85,39 | 85,08 | 84,84 | 84,95 | 85,22 | 87 | 87,78 | 84,21 | 85,27 | 85,13 | 85,27 | 84,76 | 84,45 | 84,92 | 84,81 | 84,75 | 84,77 | 84,58 | 84,86 | 89,82 | 84,92 | 85,17 | 85,19 | 94,23 | 86,94 | 87,5 | 86,77 | 86,9 | 85,74 | 86 | 88,57 | 86,82 | 92,82 | 93,75 | 100 |  |  |
| 45 | AG-Q DQ279061 | 83,07 | 83,07 | 83,07 | 82,92 | 86,95 | 82,92 | 82,23 | 86,84 | 86,35 | 82,92 | 86,44 | 82,58 | 83,05 | 83 | 86,09 | 87,39 | 86,44 | 82,89 | 87,22 | 86,84 | 82,71 | 82,74 | 83,07 | 82,95 | 83,07 | 83,1 | 82,92 | 82,74 | 86,82 | 87,3 | 88,31 | 86,84 | 87,55 | 86,65 | 86,15 | 86,67 | 85,6 | 85,56 | 89,98 | 86,65 | 91,16 | 85,67 | 86,29 | 85,81 | 100 |  |
| 46 | AG-D AB214367 | 86,19 | 86,19 | 86,19 | 85,97 | 86,7 | 85,97 | 85,91 | 86,19 | 86,1 | 86,19 | 85,75 | 85,75 | 86,19 | 86,19 | 85,75 | 86,32 | 85,97 | 86,19 | 86,61 | 86,19 | 86,19 | 86,19 | 86,19 | 86 | 86,19 | 86,44 | 85,78 | 85,97 | 86,16 | 86,71 | 87 | 85,68 | 86,36 | 85,75 | 89,41 | 89,6 | 89,86 | 90,38 | 90,87 | 90,77 | 89,57 | 88,96 | 90,25 | 89,8 | 92,57 | 100 |

Legend:

The table was prepared according to the results of the comparison by the BLAST tool in the NCBI database; sequences used in the comparisons are the same as in the phylogenetic tree; numbers in the first row correspond in order to the order of the numbers of the sequences.
